# Supplementary material for: ViSTA: A Novel Network Improving Lung Adenocarcinoma Invasiveness Prediction from Follow-Up CT Series
Source: Cancers (Basel). 2022 Jul 28;14(15):3675. doi: 10.3390/cancers14153675 (PMC9367560; doi:10.3390/cancers14153675)
Supplement: Supplementary file 1 [file cancers-14-03675-s001.zip › cancers-1783346-supplementary.pdf]

Supplementary Materials

ViSTA: A Novel Network Improving Lung Adenocarcinoma Invasiveness Prediction from Follow-Up CT Series

Section S1: Supplementary Methods

S1.1. Model Structure of ViSTA and SimTA

ViSTA consists of a CNN backbone (layers before the final fully-connected layer) and a SimTA module following the aforementioned CNN. The input of ViSTA is a series of 3D patches extracted from a sequence of follow-up visits targeted at the same nodule. These patches are first sent to the CNN backbone (ResNeXt [5] 3D in this research) to retrieve a time series of feature vectors. The feature sequence is then fed into the SimTA module [1], which captures global temporal relations using a simplified attention mechanism. A SimTA module can be made up of a single or a stack of several SimTA layers. **Supplementary Figure S1** provides an illustration of a single SimTA layer. Feature vectors from different timepoints first go through a shared linear layer and a non-linear activation. Next, an attention matrix is derived from intervals between each timepoint. The element on the i-th row and j-th column of matrix is calculated as follows:

$$A_{i,j} = \begin{cases} -\sigma(\lambda)\sum_{t=j}^{i}\tau_t + \beta, & i > j \\ 0, & i = j \\ -\infty, & i < j \end{cases}$$

Where λ and β are trainable scale and offset parameters, respectively. $\tau \in \mathbb{R}^{T-1}$ is a vector representing time intervals between two adjacent timepoints. σ represents a non-linear activation function (ReLU [2] in this research) to ensure that $-\sigma(\lambda) \leq 0$. The i-th row of represents the influence each step has over the i-th timepoint. The intention is simple and intuitive: only previous timepoints should have impact on the current one, and the earlier the timepoint is, the less influence it has. Complete mathematical formulation of SimTA layer is as follows:

$$SimTA(x) = LayerNorm(x + \sigma(w^T x + b) \otimes softmax(A))$$

where w and b are weights and biases of the linear layer, and σ is the non-linear activation function (ELU [3] is used in this research). The $\otimes$ operator represents matrix multiplication. Here the softmax function is applied over the column axis as follows:

$$softmax(A_{i,j}) = \frac{e^{A_{i,j}}}{\sum_1^T e^{A_{i,t}}}$$

where e is the natural exponent. LayerNorm [4] normalizes features over the channel dimension. After the SimTA module, the feature vector of size $T \times C$ is pooled to $1 \times C$ by keeping only the last timepoint, then it is fed into a linear layer to perform classification.

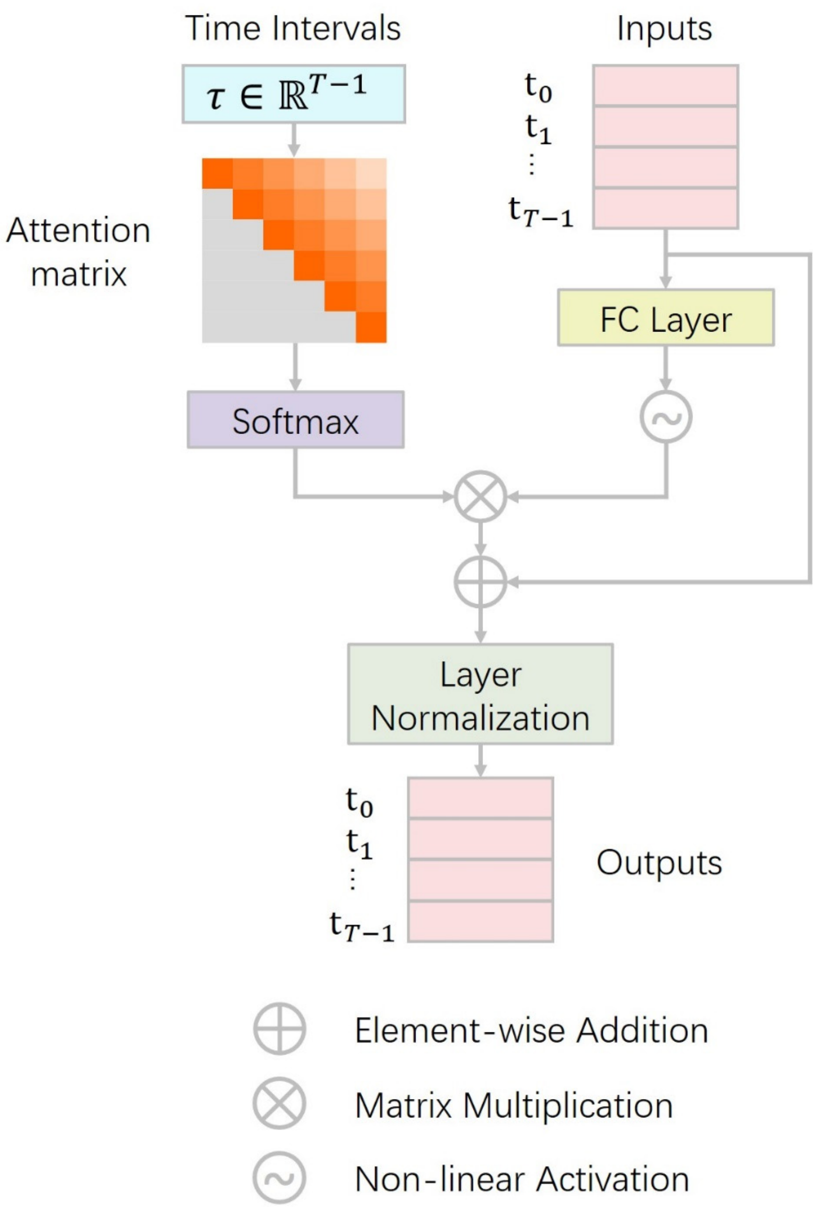

Figure S1. The illustration of a single SimTA layer. The colormap of the attention matrix indicates the magnitude of attention allocated in a particular timepoint. Gray represents zero attention. The darker the orange color is, the more attention the timepoint gets.

S1.2. VDT (Volume Doubling Time)

VDT represents the time period it takes for a nodule to double its volume. It is calculated as follows:

$$VDT = \frac{t_1 - t_0}{log_2(\frac{V_1}{V_0})}$$

where V_0 and V_1 represents the nodule volume at timepoint t_0 and t_1, respectively. Since VDT does not increase monotonously with nodule's growth ($VDT < 0$ if nodule shrinks), which is required for AUC calculation, we used $1/VDT$ in our experiments to restore the monotony. The formula of Youden index is given in Section S1.3.

S1.3 Formulas of Evaluation Metrics

To evaluate each method's performance, we used a variety of metrics including accuracy, precision, sensitivity, F1 score and AUC. Assume that TP_η, TN_η, FP_η and FN_η are true positives, true negatives, false positives and false negatives calculated at probability threshold η. The aforementioned metrics can be calculated as follows:

$$\text{accuracy}_\eta = \frac{\text{TP}_\eta + \text{TN}_\eta}{\text{TP}_\eta + \text{TN}_\eta + \text{FP}_\eta + \text{FN}_\eta}$$

$$\text{specificity}_\eta = \frac{\text{TN}_\eta}{\text{TN}_\eta + \text{FP}_\eta}$$

$$\text{sensitivity}_\eta = \frac{\text{TP}_\eta}{\text{TP}_\eta + \text{FN}_\eta}$$

$$\text{precision}_\eta = \frac{\text{TP}_\eta}{\text{TP}_\eta + \text{FP}_\eta}$$

$$F1_\eta = \frac{2 \times \text{precision}_\eta \times \text{sensitivity}_\eta}{\text{precision}_\eta + \text{sensitivity}_\eta}$$

$$\text{AUC} = \int_{\eta=0}^{1} \text{sensitivity}_\eta d\eta$$

To calculate metrics that need a cutoff value (all metrics above except for AUC), we set the threshold between IA and non-IA at the value that gives the best Youden index on the validation dataset. The calculation of Youden index is defined as:

$$Youden\ index_\eta = \text{specificity}_\eta + \text{sensitivity}_\eta - 1$$

Table S1. Different models' performances in predicting invasiveness on the training dataset. The highest among all is highlighted in bold.

	AUC	Acc.	Prec.	Sens.	F1
1/VDT (best Youden index)	64.0%	62.0%	56.2%	73.9%	63.8%
1/VDT (400 days)	64.0%	58.8%	63.2%	21.6%	32.2%
CNN last only	99.3%	95.9%	95.5%	95.5%	95.5%
CNN first only	99.7%	96.7%	98.1%	94.6%	96.3%
CNN all-first	100.0%	98.4%	96.5%	100.0%	98.2%
CNN all-last	100.0%	100.0%	100.0%	100.0%	100.0%
CNN+LSTM	100.0%	99.6%	99.1%	100.0%	99.6%
ViSTA	92.9%	80.8%	74.2%	88.3%	80.7%

Table S2. Different models' performances in predicting invasiveness on the validation dataset. The highest among all is highlighted in bold.

Heading Title	AUC	Acc.	Prec.	Sens.	F1
1/VDT (best Youden index)	62.4%	56.8%	51.9%	82.4%	63.6%
1/VDT (400 days)	62.4%	51.4%	42.9%	17.7%	25.0%
CNN last only	74.4%	67.6%	64.7%	64.7%	64.7%
CNN first only	67.4%	73.0%	81.8%	52.9%	64.3%
CNN all-first	75.3%	73.0%	70.6%	70.6%	70.6%
CNN all-last	76.8%	75.7%	78.6%	64.7%	71.0%
CNN+LSTM	79.1%	73.0%	66.7%	82.4%	73.7%
ViSTA	85.0%	78.4%	69.6%	94.1%	80.0%

References

1. Yang, J.; Chen, J.; Kuang, K.; Lin, T.; He, J.; Ni, B. MIA-Prognosis: A Deep Learning Framework to Predict Therapy Response. In Proceedings of the International Conference on Medical Image Computing and Computer-Assisted Intervention, Lima, Peru, 4–8 October 2020.
2. Krizhevsky, A.; Sutskever, I.; Hinton, G. ImageNet Classification with Deep Convolutional Neural Networks. *Commun. ACM* **2017**, *60*, 84–90. https://doi.org/10.1145/3065386.
3. Clevert, D.-A.; Unterthiner, T.; Hochreiter, S. Fast and Accurate Deep Network Learning by Exponential Linear Units (ELUs). *arXiv* **2015**, arXiv:1511.07289. https://doi.org/10.48550/arXiv.1511.07289.
4. Ba, J.L.; Kiros, J.R.; Hinton, G.E. Layer Normalization. *arXiv* **2016**, arXiv:1607.06450.
5. Xie, S.; Girshick, R.; Dollár, P.; Tu, Z.; He, K. Aggregated Residual Transformations for Deep Neural Networks. In Proceedings of the IEEE Conference on Computer Vision and Pattern Recognition (CVPR), Honolulu, HI, USA, 21–26 July 2017.
